# Supplementary material for: Venomix: a simple bioinformatic pipeline for identifying and characterizing toxin gene candidates from transcriptomic data
Source: PeerJ. 2018 Jul 31;6:e5361. doi: 10.7717/peerj.5361 (PMC6074769; doi:10.7717/peerj.5361)
Supplement: Supplemental Information 6 [file peerj-06-5361-s006.gz › FinalOutput_1E-6/L-amino-acid_oxidase_31/finaltree.pdf]

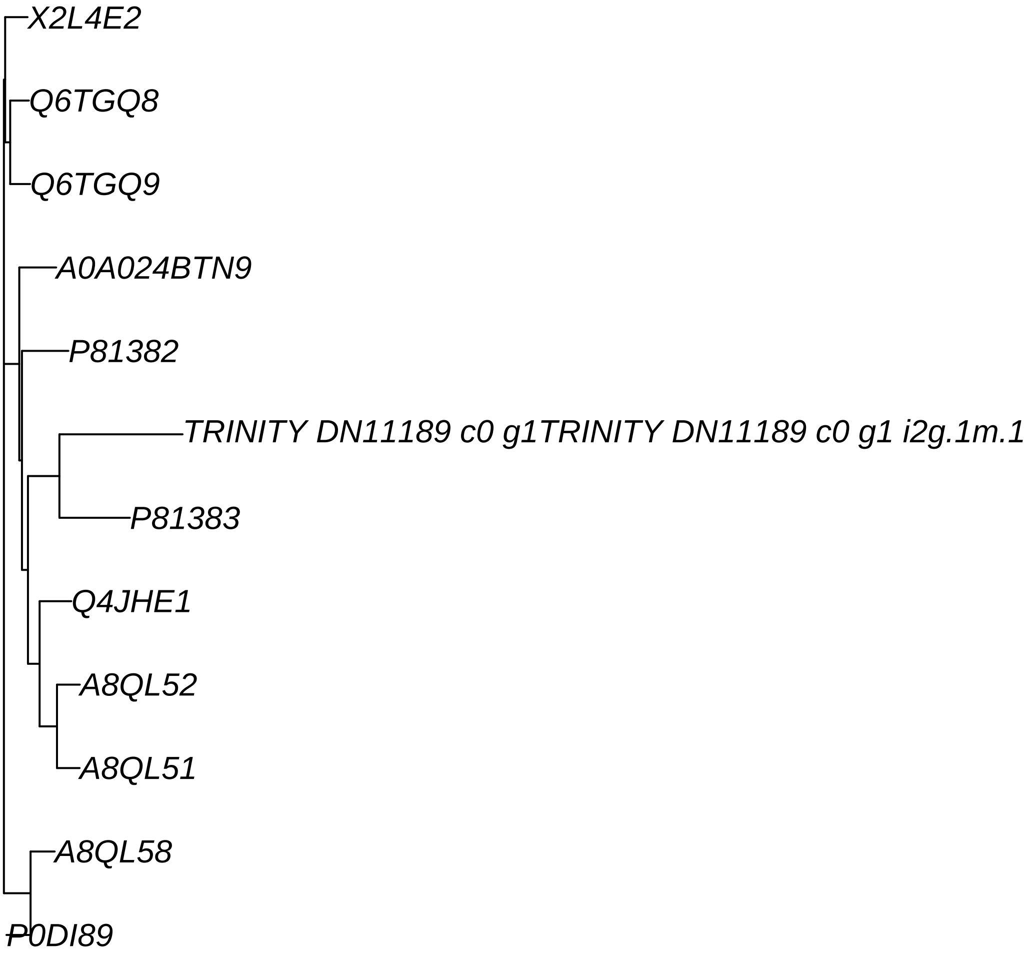
X2L4E2
Q6TGQ8
Q6TGQ9
A0A024BTN9
P81382
TRINITY DN11189 c0 g1TRINITY DN11189 c0 g1 i2g.1m.1
P81383
Q4JHE1
A8QL52
A8QL51
A8QL58
P0DI89
